# The Association Between Egg and Egg-Derived Cholesterol Consumption, and Their Change Trajectories, with Obesity Among Chinese Adults: Results from the China Health and Nutrition Survey

**DOI:** 10.3390/nu17020333

**Published:** 2025-01-17

**Authors:** Tianhui Tang, Binghua Chen, Jiahao Hu, Hangzhao Fan, Zilan Zhang, Tianyang Zhai, Chunxiao Li, Duolao Wang, Wanli Xue, Leilei Pei, Fangyao Chen, Baibing Mi, Yaling Zhao

**Affiliations:** 1 Department of Epidemiology and Biostatistics, School of Public Health, Xi’an Jiaotong University Health Science Center, No. 76, Yanta West Road, Xi’an 710061, China; 2 MRC Epidemiology Unit, Institute of Metabolic Science, University of Cambridge, Cambridge Biomedical Campus, Cambridge CB2 0QQ, UK; 3 Department of Clinical Sciences, Liverpool School of Tropical Medicine, Pembroke Place, Liverpool L3 5QA, UK; 4 Department of Nutrition and Food Safety, School of Public Health, Xi’an Jiaotong University Health Science Center, Xi’an 710061, China

**Keywords:** CHNS, eggs, dietary cholesterol, change pattern, general obesity, central obesity

## Abstract

**Background/Objectives:** As a widely consumed, nutritious, and affordable food, eggs and their derivatives’ impacts on obesity remain inconclusive. In this study, we aimed to determine the association between egg and egg-derived cholesterol consumption, and their change trajectories, with obesity among Chinese adults. **Methods**: Longitudinal data collected by the China Health and Nutrition Survey from 1997 to 2015 were analyzed. The latent growth mixture model was used to identify eggs and egg-derived cholesterol consumption trajectories. Cox proportional hazard models with shared frailty were used to analyze the association between egg and egg-derived cholesterol consumption, and their change trajectories, with obesity. **Results**: Data from 10,971 and 9483 participants aged ≥18 years old were used for the analyses of general obesity and central obesity, respectively. Compared to participants with an average egg intake of 0.1–50.0 g/d during the follow-up period, adults who never consumed eggs or those with an average egg intake of 50.1–100.0 g/d and >100.0 g/d had a higher risk of general obesity, with hazard ratios (HRs) and 95% confidence intervals (CIs) of 1.31 (1.08, 1.58), 1.30 (1.07, 1.60), and 1.98 (1.17, 3.35), respectively, and had a higher risk of central obesity, with HRs (95% CIs) of 1.17 (1.04, 1.31), 1.31 (1.14, 1.50), and 1.64 (1.15, 2.36), respectively. Participants with a “Baseline Low-Significant Rising Pattern” or a “Baseline High-Rising then Falling Pattern” of egg consumption trajectories during the follow-up period had a higher risk of general obesity, with HRs (95% CIs) of 1.56 (1.25, 1.93) and 1.38 (1.13, 1.69), respectively, and central obesity, with HRs (95% CIs) of 1.47 (1.29, 1.68) and 1.52 (1.34, 1.72), respectively. Compared to the second quartile (*Q*_2_) group of the average egg-derived cholesterol intake during the follow-up period, *Q*_1_, *Q*_3_, and *Q*_4_ groups had a higher risk of general obesity, with HRs (95% CIs) of 1.28 (1.06,1.54), 1.21 (1.02, 1.44), and 1.43 (1.19, 1.71), respectively, and a higher risk of central obesity, with HRs (95% CIs) of 1.20 (1.08, 1.33), 1.11 (1.01, 1.23), and 1.32 (1.19, 1.46), respectively. Participants with a “Baseline Low-Significant Rising Pattern” or with a “Baseline High-Rising then Falling Pattern” of egg-derived cholesterol consumption during the follow-up period had a higher risk of general obesity, with HRs (95% CIs) of 1.54 (1.25, 1.92) and 1.37 (1.15, 1.64), respectively, and a higher risk of central obesity, with HRs (95% CIs) of 1.46 (1.28, 1.68) and 1.47 (1.32, 1.64), respectively. **Conclusions**: Both the insufficient and excessive intake of eggs and egg-derived cholesterol tended to be associated with a higher risk of general and central obesity. Suddenly increasing or consistently high levels of egg and egg-derived cholesterol intake seemed to be associated with a higher risk of obesity. To prevent obesity, people should consume a moderate amount of eggs and egg-derived cholesterol.

## 1. Introduction

Obesity, characterized by excessive fat accumulation and impaired energy homeostasis, is a complex health issue arising from genetic, environmental, and lifestyle factors [1]. Obesity has become a major global public health challenge, and the prevalence of obesity has nearly doubled in recent decades [2,3]. Studies showed that obesity was a risk factor for many kinds of non-communicable diseases, such as, type 2 diabetes, cardiovascular diseases, heart disease, and cancers [2,3]. Obesity and obesity-related complications have caused severe health threats and diseases and have become significant health problems in both developed and developing countries [4].

Identifying modifiable risk factors is crucial for the prevention and control of obesity. As a widely consumed, nutritious, and affordable food, eggs and their derivatives’ impacts on human health have been widely discussed [5]. However, the results of the studies on the association between egg consumption and obesity are controversial. The results of cross-sectional studies found that egg intake was positively associated with body mass index (BMI) and waist circumference (WC) in US adults [6] and positively associated with WC among Chinese adults [7]. But results from three US cohorts showed that egg intake was not associated with BMI [8]. A meta-analysis of clinical trials also reported that egg intake was not associated with BMI and WC [9]. Studies in China and Korea reported that egg intake was negatively associated with central obesity [10,11].

Although eggs are an important and affordable source of high-quality protein, iron, unsaturated fatty acids, phospholipids, and carotenoids, questions remain about the benefits and risks of eating eggs regularly [12]. The major concern of egg consumption is about the high cholesterol content in eggs. One egg contains approximately 200~600 mg of cholesterol [13,14] and cholesterol from eggs is the main source of dietary cholesterol [13]. The relationship between dietary cholesterol and obesity is still controversial. Some studies found that cholesterol from egg yolk may influence fat accumulation and metabolic dysfunction [15], while others reported that dietary cholesterol has limited impact on obesity when consumed within the recommended levels [16]. Given the high prevalence of obesity [3] and the widespread consumption of eggs worldwide [17], clarifying the role of egg-derived cholesterol in the development of obesity could have significant implications for dietary interventions aimed at weight management and metabolic health.

So far, the association between egg and egg-derived cholesterol consumption and obesity remains inconclusive. The long-term effects of egg and egg-derived cholesterol consumption, and their change trajectories, on obesity have not been estimated. In this study, using data from a large nationwide longitudinal study, the China Health and Nutrition Survey (CHNS), we aimed to examine the association between egg and egg-derived cholesterol intake, and their change trajectories, with obesity among Chinese adults. We hypothesized that moderate and stable egg and egg-derived cholesterol intake would be beneficial for the prevention of obesity.

## 2. Materials and Methods

### 2.1. Study Design and Participants

In this study, we used data collected by the CHNS. The CHNS is an open ongoing prospective longitudinal study that aims to collect representative data on critical public health risk factors, health outcomes, and the nutritional status of the Chinese population [18]. Details about the design and procedures of the CHNS have been reported elsewhere [18,19].

**Figure 1:**
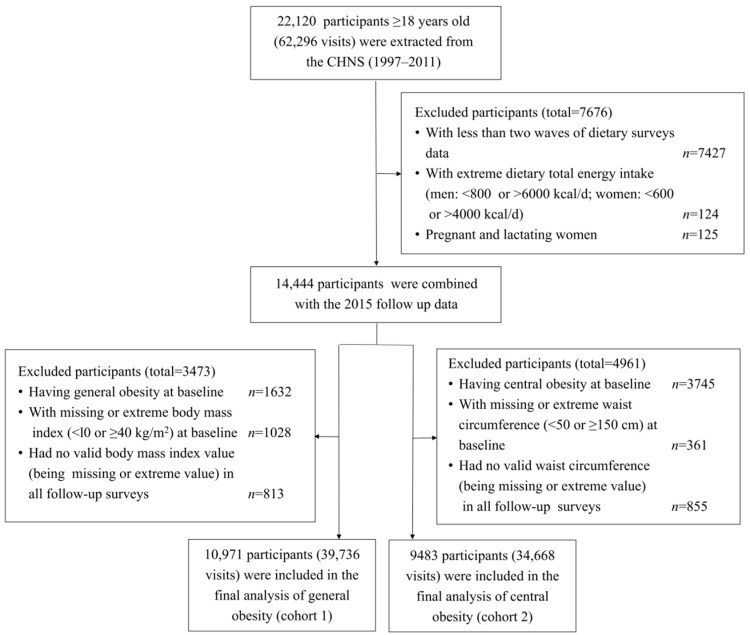
Flow diagram of participants in the study.

Our study used data from seven waves of the CHNS (1997–2015). Dietary data from the 2015 wave has not yet been opened to the public, so we only used the health outcome information from 2015. Participants aged <18 years old at baseline had less than two waves of dietary data and had an extreme total dietary energy intake (<800 kcal/d or >6000 kcal/d for men, <600 kcal/d or >4000 kcal/d for women) [20], and pregnant and lactating women were excluded from our analyses. In addition, participants who had general obesity (BMI ≥ 28.0 kg/m^2^) or had a missing or extreme BMI value (BMI < 10.0 kg/m^2^ or ≥40.0 kg/m^2^) at baseline or had no valid BMI (either missing or extreme) in all follow-up surveys they participated in were excluded from the analyses of general obesity. Participants who had central obesity (WC ≥ 85.0 cm for women and ≥90.0 cm for men) or had a missing or extreme WC (WC < 50.0 cm or ≥150.0 cm) at baseline or had no valid WC (either missing or extreme) in all follow-up surveys they participated in were excluded from the analyses of central obesity. Finally, a total of 10,971 and 9483 participants were included in the analyses of general obesity (cohort 1, with 39,736 visits) and central obesity (cohort 2, with 34,668 visits), respectively. The selection process for the study participants is presented in Figure 1.

### 2.2. Definition of Follow-Up in the Study

Participants in this study were prospectively followed from the time of their first visit to the CHNS. The CHNS is an open cohort. Participants could join or leave the cohort at any wave of the survey. Participants who were lost to follow-up in one wave could rejoin the survey in subsequent waves. We defined the baseline of the participants as the time when they first participated in the CHNS between 1997 and 2011. The follow-up duration was defined as the period from the first visit to the CHNS to the latest visit that the participant attended or the first occurrence of the outcome (general obesity or central obesity), death, or loss to follow-up from the CHNS.

### 2.3. Exposure Assessment

Exposure variables in this study included egg and egg-derived cholesterol intake and the change trajectories of egg and egg-derived cholesterol intake during the follow-up period. In each wave of the CHNS, three consecutive 24 h dietary recalls, including two weekdays and one weekend day, were used to collect dietary intake data at the individual level, and household food consumption data during the same three-day period were also collected. Each individual’s egg intake (g/d) was collected by the three consecutive 24 h dietary recalls. Egg-derived cholesterol intake (mg/day) was calculated based on egg consumption and the China Food Composition Table 2002 [14], i.e., using the following formula: egg intake (g) × 0.88 × cholesterol content (mg)/100 (g), where 0.88 represents the fraction of the edible portion of an egg and cholesterol content (mg)/100 (g) represents the amount of cholesterol per 100 g edible portion of different types of eggs. The identification of the change trajectories of egg and egg-derived cholesterol intake of the participants is presented in the Statistical Analyses section.

### 2.4. Outcome Ascertainments

The main outcomes of interest in this study were general obesity and central obesity. According to the recommendation of the China Obesity Working Group in 2002 [21], general obesity was defined as BMI ≥ 28.0 kg/m^2^, and central obesity was defined as WC ≥ 85.0 cm for women and ≥90.0 cm for men in our analyses. Following standardized procedures, the weight and height of all participants were measured by trained health workers using calibrated equipment (SECA 880 scales and SECA 206 wall-mounted metal tapes, SECA GmbH & Co. KG, Hamburg, Germany) [22,23]. BMI was calculated as weight (kg) divided by the square of height (m^2^). WC was measured at a point midway between the lowest edge of the rib cage and the highest edge of the iliac crest in a horizontal plane using nonelastic tape [24]. The primary outcome was the time from baseline to the first occurrence of general or central obesity during the follow-up.

### 2.5. Assessments of Covariates

Participants’ sociodemographic characteristics (including gender, age, nationality, education level, marital status, family economic level, community type, and region), lifestyle factors (including smoking, drinking, and physical activity), and history of diseases were collected with the questionnaire in each wave of the CHNS. Nationality was categorized as Han and other nationalities. Education levels were divided into four categories: illiteracy, primary school, middle school, and high school and above. Marital status was categorized into three groups: married, unmarried, and divorced/separate/widowed. Participants’ per capita annual family income at baseline was divided into three levels (high, middle, and low) according to the per capita annual family income tertiles. Community type included four categories: city, suburb, town, and village. The region was categorized into four categories: Northeast (Heilongjiang and Liaoning provinces), East Coast (Shandong and Jiangsu provinces), Central (Henan, Hubei, and Hunan provinces), and Western (Guangxi autonomous region and Guizhou province). Participants were divided into current smokers and non-smokers, and current drinkers and non-drinkers, according to their current smoking and drinking status, respectively. Participants’ physical activity levels were classified into three categories (light, medium, and heavy) based on their self-reported activities, including occupational, domestic, transportation, and leisure-time physical activities. History of stroke, myocardial infarction, and diabetes were self-reported by the participants. Participants’ dietary total energy intake, meat intake, and dietary total protein intake were calculated by using dietary intake information collected by the 24 h dietary recalls and household food consumption data and the Chinese Food Composition Table [14].

### 2.6. Statistical Analyses

Continuous variables were presented as mean ± standard deviation (SD). Categorical variables were presented as frequencies (%). Analyses of variance and Chi-square tests were used to compare continuous and categorical variables, respectively. Assuming an egg weighs 50 g, participants were categorized into four groups according to convenient cut-off values of average daily egg consumption during the follow-up period: 0.0 g/d, 0.1–50.0 g/d, 50.1–100.0 g/d, and >100.0 g/d. Egg-derived cholesterol intake (mg/d) was divided into four categories according to its quartiles (*Q*_1_ to *Q*_4_).

We used the latent growth mixture model (LGMM) to identify the change trajectories of egg and egg-derived cholesterol intake of participants, respectively. The LGMM is a maximum-likelihood parameter estimation method to reveal the trajectories for repeated measure metrics, identifying homogeneous subgroups with similar trajectory patterns over time and permitting variance within classes [25]. We fitted the LGMM with one to five trajectory classes and determined the optimal trajectory class model according to the following criteria: (a) Bayesian information criterion (BIC), with a smaller value indicating a better fit of the model; (b) the number of people assigned to each trajectory should include at least 5% of the total population; and (c) the mean posterior probability of each trajectory class should be greater than 70%. According to the trajectories estimated by the LGMM, each participant was assigned to the trajectory pattern with the highest probability.

Cox proportional hazards regression models with shared frailty were used to estimate the hazard ratios (HRs) and 95% confidence intervals (CIs) for the associations of egg intake, egg-derived cholesterol consumption and their change trajectory patterns, respectively, with obesity. Model 1 used the average intake of eggs, egg-derived cholesterol consumption, and their change trajectory patterns during the follow-up period, respectively, as the fixed effect term, and family as the random effect term. In model 2, we made further adjustments for participants’ sociodemographic factors (including gender, age, nationality, education levels, marital status, family economic level, community type, and region), lifestyle factors (including smoking, drinking, and physical activity), dietary intake (dietary total energy intake, meat intake, and dietary total protein intake), history of diseases (including stroke, myocardial infarction, and diabetes), and the baseline year.

In the subgroup analyses, in addition to the main effects, we also evaluated the potential effect of modification through a likelihood ratio test, which compared models with and without the interaction terms between stratification variables (e.g., gender, age, smoking, drinking, and physical activity) and all exposure variables to determine whether the association between eggs and egg-derived cholesterol and obesity differed across the subgroups. We also conducted two kinds of sensitivity analyses to evaluate the robustness of our findings. First, we excluded participants who had less than 3 dietary measurements and conducted the analyses among those who had ≥3 dietary measurements. Second, to evaluate the effect of missing covariates data on the association between exposure variables and obesity, we performed sensitivity analyses on participants with missing values imputed by using the multiple imputation method. Sociodemographic factors, lifestyle factors, dietary intake, history of diseases, and the baseline year were adjusted for in all the subgroup and sensitivity analyses.

Statistical analyses were performed by using R 4.3.1 (R Studio Inc., Boston, MA, USA). All statistical tests were two-tailed, and statistical significance was set at *p* < 0.05.

## 3. Results

### 3.1. General Characteristics of Participants in the Study

The data of a total of 10,971 participants with 39,736 visits (cohort 1) were used for the study of general obesity. The participants were 42.33 ± 15.33 years old at baseline and 52.3% of them were women. They visited 3.62 ± 1.57 times and were followed for 8.87 ± 5.19 years. In the study of central obesity (cohort 2), the data of a total of 9483 participants with 34,668 visits were analyzed. The participants were 41.29 ± 15.12 years old at baseline and 52.0% of them were women. They visited 3.66 ± 1.81 times and were followed 7.56 ± 4.73 years. The general characteristics of participants at baseline are presented in Table 1.

### 3.2. Trajectory Patterns of Egg and Egg-Derived Cholesterol Consumption of Participants

**Figure 2:**
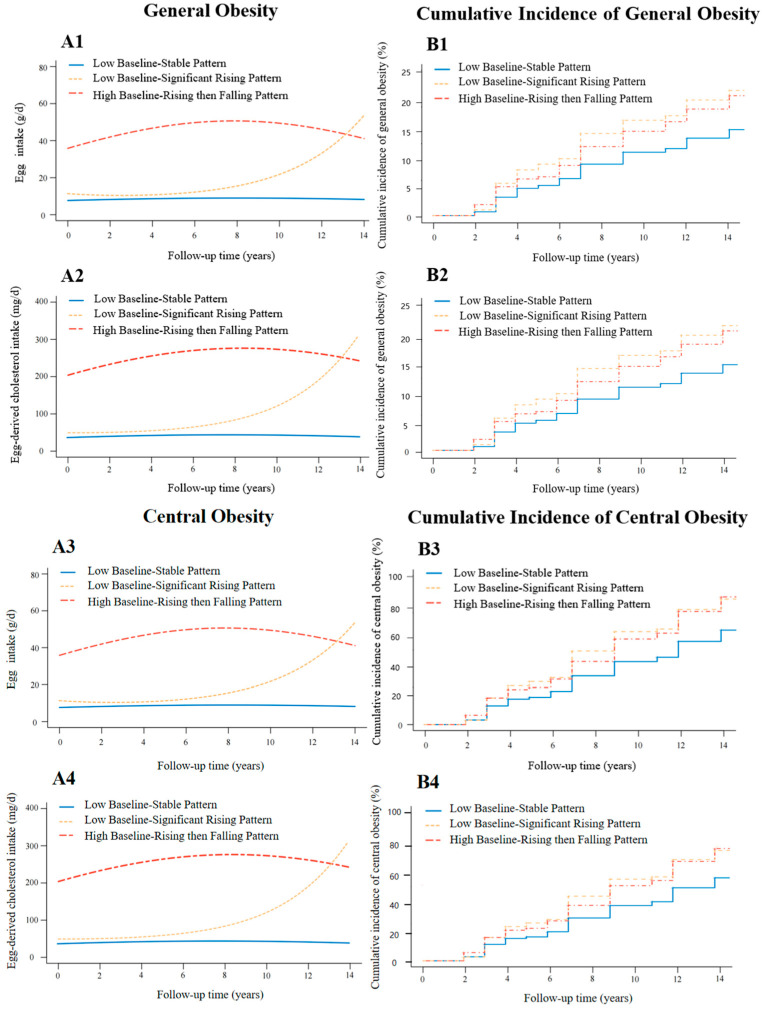
Change trajectories of egg and egg-derived cholesterol consumption and the cumulative incidence of general obesity and central obesity among participants in this study. (**A1**,**A2**): change trajectories of egg and egg-derived cholesterol intake, respectively, among participants in cohort 1 for the analysis of general obesity. (**A3**,**A4**): change trajectories of egg and egg-derived cholesterol intake, respectively, among participants in cohort 2 for the analysis of central obesity. (**B1**,**B2**): cumulative incidence of general obesity of different patterns of change trajectory of egg and egg-derived cholesterol intake, respectively, among participants in cohort 1. (**B3**,**B4**): cumulative incidence of central obesity of different patterns of change trajectory of egg and egg-derived cholesterol intake, respectively, among participants in cohort 2.

According to the baseline level and change speed of egg and egg-derived cholesterol intake, three patterns of egg and egg-derived cholesterol intake change trajectory were identified among participants in cohort 1 for the analysis of general obesity and among participants in cohort 2 for the analysis of central obesity, respectively (Figure 2(A1–A4)). The general characteristics of participants in each trajectory group at baseline are presented in Supplementary Tables S1 and S2.

In the study of general obesity (cohort 1), 3438 (31.3%) participants characterized by stably consuming around 7.7 g/d of eggs (49.5 mg/d of egg-derived cholesterol) during the follow-up period were classified into the “Low Baseline-Stable Pattern”. A total of 1715 (15.6%) participants characterized by an increase in egg consumption from 11.1 g/d to 53.5 g/d (from 49.5 mg/d to 300.0 mg/d of egg-derived cholesterol consumption) during the follow-up period were classified into the “Low Baseline-Significant Rising Pattern”. A total of 5818 (53.0%) participants characterized by an increase in egg consumption from 35.8 g/d to 50.8 g/d (from 200.0 mg/d to 266.0 mg/d of egg-derived cholesterol consumption) within the first eight years of follow-up and then a decline of egg consumption to 41.0 g/d (235.0 mg/d of egg-derived cholesterol) within the subsequent six years of follow-up were classified into the “High Baseline-Rising then Falling Pattern” (Figure 2(A1)). As participants’ egg-derived cholesterol intake values (mg/d) were calculated based on their egg intake levels, the patterns of change trajectories of egg-derived cholesterol consumption and population composition for each trajectory pattern were the same as those of egg consumption (Figure 2(A2)).

The trajectory patterns of egg and egg-derived cholesterol consumption of participants in the study of central obesity (cohort 2) are similar. A total of 3102 (32.7%) participants characterized by stably consuming around 8.4 g/d of eggs (40.0 mg/d of egg-derived cholesterol) during the follow-up period were classified into the “Low Baseline-Stable Pattern”. A total of 1530 (16.1%) participants characterized by an increase in egg consumption from 11.4 g/d to 50.6 g/d (from 48.3 mg/d to 295.0 mg/d of egg-derived cholesterol consumption) during the follow-up period were classified into the “Low Baseline-Significant Rising Pattern”. A total of 4851 (51.2%) participants characterized by an increase in egg consumption from 34.1 g/d to 50.0 g/d (from 200.0 mg/d to 258.0 mg/d of egg-derived cholesterol consumption) within the first eight years of follow-up and then a decline of egg consumption to 38.2 g/d (220.0 mg/d of egg-derived cholesterol) within the subsequent six years of follow-up were classified into the “High Baseline-Rising then Falling Pattern” (Figure 2(A3)). Figure 2(A4) presents the patterns of change trajectories of egg-derived cholesterol consumption and population composition for each trajectory pattern.

### 3.3. Association Between Egg Consumption and Its Change Trajectories and the Risk of Obesity

In the analysis of general obesity (cohort 1), during the average follow-up period of 8.87 years, 1150 (10.5%) participants became generally obese. After being adjusted for sociodemographic factors, lifestyle factors, dietary total energy intake, meat intake, history of stroke, myocardial infarction and diabetes, and the baseline year, the results of model 2 showed that, compared with participants who averagely consumed 0.1–50.0 g/d of eggs during the follow-up period, participants who did not consume eggs, consumed 50.1–100.0 g/d of eggs, and consumed more than 100.0 g/d of eggs had a higher risk of general obesity. The HRs (95% CIs) were 1.31 (1.08, 1.58), 1.30 (1.07, 1.60), and 1.98 (1.17, 3.35), respectively (Table 2).

In the analysis of central obesity (cohort 2), during the average follow-up period of 7.56 years, 3443 (31.0%) participants became centrally obese. After being adjusted for the confounders, the results of model 2 showed that, compared to the participants who averagely consumed 0.1–50.0 g/d of eggs during the follow-up period, participants who did not consume eggs, consumed 50.1–100.0 g/d of eggs, and consumed more than 100.0 g/d of eggs had a higher risk of central obesity. The HRs (95% CIs) were 1.17 (1.04, 1.31), 1.31 (1.14, 1.50), and 1.64 (1.15, 2.36), respectively (Table 2).

The risk of general obesity in adults with different patterns of egg consumption trajectories among participants in cohort 1 are presented in Table 2 and Figure 2(B1). Compared to the “Low Baseline-Stable Pattern”, the results of model 2 showed that participants with the “Low Baseline-Significant Rising Pattern” and “High Baseline-Rising then Falling Pattern” of egg consumption had a higher risk of general obesity (HRs (95% CIs): 1.56 (1.25, 1.93) and 1.38 (1.13, 1.69)).

The risk of central obesity in adults with different patterns of egg consumption trajectory among participants in cohort 2 are presented in Table 2 and Figure 2(B3). Compared to the “Low Baseline-Stable Pattern”, the results of model 2 showed that participants with the “Low Baseline-Significant Rising Pattern” and “High Baseline-Rising then Falling Pattern” of egg consumption had a higher risk of central obesity (HRs (95% CIs): 1.47 (1.29, 1.68) and 1.52 (1.34, 1.72)).

### 3.4. Association Between Egg-Derived Cholesterol Consumption and Its Change Trajectories and the Risk of Obesity

The results of the association between average egg-derived cholesterol consumption during the follow-up period and the risk of general and central obesity are presented in Table 3. After being adjusted for the sociodemographic factors, lifestyle factors, dietary total energy intake, dietary total protein intake, history of stroke, myocardial infarction and diabetes, and the baseline year, the results of model 2 showed that, compared to the participants in the *Q*_2_ group of egg-derived cholesterol consumption, participants in the *Q*_1_, *Q*_3_, and *Q*_4_ groups had a higher risk of general obesity. The HRs (95% CIs) were 1.28 (1.06, 1.54), 1.21 (1.02, 1.44), and 1.43 (1.19, 1.71) for the *Q*_1_, *Q*_3_, and *Q*_4_ groups, respectively. Similarly, compared to the *Q*_2_ group of egg-derived cholesterol consumption, the *Q*_1_, *Q*_3_, and *Q*_4_ groups had higher hazards of central obesity. The HRs (95% CIs) were 1.20 (1.08, 1.33), 1.11 (1.01, 1.23) and 1.32 (1.19, 1.46), respectively.

Hazards of general and central obesity among adults with different patterns of egg-derived cholesterol consumption trajectory among participants are presented in Table 3 and Figure 2(B2,B4). The results of model 2 showed that compared to the “Low Baseline-Stable Pattern”, participants with the “Low Baseline-Significant Rising Pattern” and “High Baseline-Rising then Falling Pattern” of egg-derived cholesterol consumption had a higher risk of general obesity (with HRs (95% CIs) of 1.54 (1.25, 1.92) and 1.37 (1.15, 1.64)) and central obesity (with HRs (95% CIs) of 1.46 (1.28, 1.68) and 1.47 (1.32, 1.64)).

### 3.5. Subgroup Analyses and Sensitivity Analyses

The results of the subgroup analyses by gender, age, smoking, drinking, and physical activity status are presented in Supplementary Tables S3–S6. The association between egg and egg-derived cholesterol intake, and their change trajectories, with obesity were similar among the subgroups (all *p*-interaction values were greater than 0.05). We did not observe evidence of differences in the risk of obesity associated with eggs and egg-derived cholesterol across subgroups defined by gender, age, smoking, drinking, and physical activity status. The results of the two kinds of sensitivity analyses, which are the analyses among participants with at least three dietary measurements or among participants with missing values in covariates imputed by using the multiple imputation method, did not differ very much from the results of the primary analytic samples, with the direction and magnitude of the association being persistent (Supplementary Tables S7 and S8).

## 4. Discussion

By using longitudinal data from the CHNS, our present study assessed the association between egg and egg-derived cholesterol consumption, and their change trajectories, with the risks of general and central obesity. The results showed that, compared to participants who on average consumed 0.1–50.0 g/d of eggs during the follow-up period, both never consuming eggs and consuming over 50.0 g/d of eggs were associated with a higher risk of general and central obesity. Compared to the *Q*_2_ group, individuals in the *Q*_1_, *Q*_3_, and *Q*_4_ groups of egg-derived cholesterol consumption had a higher risk of general and central obesity. Our study found that a sudden increase or consistently maintaining high levels of egg and egg-derived cholesterol intake was associated with higher risks of general and central obesity

The results of our study suggest that the moderate intake of eggs is beneficial to the prevention of obesity, that is, people should consume one egg per day, rather than never eating eggs or eating more than one egg per day. In a cross-sectional study of 2241 Chinese adults aged 18–80 years, Liu et al. found that maintaining a certain intake of eggs could reduce the risk of central obesity and the percentage of body fat [10], which is consistent with the findings of our study. The biological mechanisms by which moderate egg intake may prevent obesity are not well understood. Eggs are an important food source to meet the daily protein needs of Chinese people [26]. Studies have shown that egg intake could improve satiety and reduce food intake within 24 h [27]. A meta-analysis showed that high-protein diets could reduce energy intake by regulating energy metabolism and appetite, thereby maintaining long-term weight stability [28]. Additionally, consuming egg-derived protein may stimulate the production of anorexic hormones, such as cholecystokinin, which has been found to have an effect on regulating food intake [29]. However, although high-protein diets can enhance satiety and reduce overall calorie intake, studies have shown that the overconsumption of eggs, often accompanied by high-fat and high-sugar foods, and could disrupt the gut microbiota balance, affect energy extraction and storage, and increase fat accumulation [30,31]. Therefore, while eggs can be part of a healthy diet, they should be consumed in moderation to avoid the potential risk of weight gain.

Our study found that both lower and higher egg-derived cholesterol consumption were associated with higher risks of general and central obesity. Non-high-density lipoprotein cholesterol (non-HDL-C), a comprehensive marker that includes low-density lipoprotein cholesterol (LDL-C), very low-density lipoprotein cholesterol (VLDL-C), and other atherogenic lipoproteins, has emerged as a predictor of metabolic disorders [32]. Although the direct effects of egg-derived cholesterol on non-HDL-C remain underexplored, some studies have reported that a high dietary cholesterol intake may lead to modest increases in non-HDL-C concentrations [33]. Oxidized non-HDL-C particles may contribute to obesity through mechanisms such as lipid peroxidation, systemic inflammation, and impaired lipid clearance [34]. Studies showed that dietary cholesterol intake, including that from eggs, could increase the oxidation susceptibility of LDL, and enhance the adverse effects of saturated fatty acids (SFAs) [35]. Oxidized LDL particles are positively associated with increased risks of obesity, diabetes, and metabolic diseases [36]. However, eggs are also rich in antioxidant carotenoids, such as lutein and zeaxanthin, which can reduce LDL oxidation [35]. These beneficial components may counteract the negative effects of egg-derived cholesterol, suggesting that the moderate consumption of egg-derived cholesterol does not necessarily lead to obesity. Our study showed that participants in the lowest quartile (*Q*_1_) of egg-derived cholesterol intake had a higher risk of general and central obesity. This may be due to the low intake of eggs and egg-derived protein, which increases the risk of obesity. Furthermore, dietary cholesterol consumption is often accompanied by a high intake of SFAs [7]. SFAs may contribute to obesity through mechanisms involving inflammation [37], enhanced fat storage [38], and insulin resistance [39]. The adverse effects of high egg-derived cholesterol intake on obesity may be partly attributed to the high SFAs intake that accompanies high egg-derived cholesterol intake.

Our study found that a sudden increase or consistently maintaining high levels of egg and egg-derived cholesterol intake was associated with higher hazards of general and central obesity. One possible mechanism is that a sudden increase or maintenance of high levels of egg and egg-derived cholesterol intake may lead to excessive calorie consumption and subsequent weight gain, which could disturb metabolic homeostasis and trigger a vicious cycle of fat accumulation and further weight gain [40]. This process can increase metabolic stress, elevate insulin resistance, and promote lipid storage, ultimately exacerbating obesity [41]. Additionally, metabolic overload may disrupt lipid distribution, contributing to central obesity, which is closely linked to an increased risk of cardiovascular diseases [42].

Our study had several strengths. First, our study is based on the prospective cohort data collected by the CHNS. The participants of the CHNS are a large nationally representative prospective longitudinal sample of Chinese adults. Second, the data of height, weight, and WC used in our study were measured by investigators rather than participants’ self-reported data. This ensured the accuracy and reliability of the primary outcome variables of our study. Third, with the LGMM, we extensively used the longitudinal data to estimate patterns of the change trajectories of the egg and egg-derived cholesterol consumption and provided evidence of the association between long-term egg and egg-derived cholesterol consumption and the hazards of general and central obesity.

Our study also had several limitations. First, individuals’ dietary data in the CHNS were collected with three consecutive 24 h dietary recalls. Although the 24 h dietary recall is a commonly used dietary assessment tool, it is not an ideal method for assessing an individual’s long-term dietary habits. However, the 24 h dietary recall is suitable for assessing the dietary consumption of a population with a large sample size [43] and was suitable to assess the change trajectories of eggs and egg-derived cholesterol and their association with general and central obesity. Second, although we made adjustments for many confounding factors in the present analyses, some unmeasured potential confounders, such as genetic traits and poultry rearing practices, and cooking methods of eggs were not controlled for. Cooking methods may influence the digestion and absorption of food and then affect the association of egg consumption and health outcomes. Third, our study was based on data from Chinese adults, so caution should be taken when generalizing the results to other populations. Finally, it is necessary to emphasize that our study is an observational study based on an opening cohort. This limited us from establishing definitive causal conclusions. Further interventional studies are needed to verify the relationship between egg consumption and obesity.

## 5. Conclusions

Our study found that either the insufficient or excessive intake of eggs and egg-derived cholesterol was associated with a higher risk of general and central obesity. A sudden increase in or maintenance of a consistently high intake of eggs and egg-derived cholesterol was also associated with higher risks of obesity. The long-term and consistent intake of moderate amounts of eggs and egg-derived cholesterol is beneficial for the prevention of obesity.

## Figures and Tables

**Table 1:** General characteristics of participants at baseline in the study.

Characteristics	General Obesity			Central Obesity		
	No	Yes	Total	No	Yes	Total
	(*n* = 9821, 89.5%)	(*n* = 1150, 10.5%)	(*n* = 10,971)	(*n* = 6040, 69.0%)	(*n* = 3443, 31.0%)	(*n* = 9483)
Duration of follow-up, years, Mean ± SD	9.14 ± 5.23	6.61 ± 4.25	8.87 ± 5.19	8.26 ± 4.98	6.35 ± 3.96	7.56 ± 4.73
Egg intake, g/d, Mean ± SD	21.86 ± 29.99	24.76 ± 31.41	22.16 ± 30.15	19.72 ± 28.40	22.16 ± 30.01	20.61 ± 29.02
Egg-derived cholesterol intake, mg/d, Mean ± SD	124.66 ± 171.02	141.19 ± 179.11	126.39 ± 171.95	112.48 ± 161.94	126.38 ± 171.14	117.52 ± 165.46
Men, *n* (%)	4702 (47.9)	531 (46.2)	5233 (47.7)	3044 (50.4)	1505 (43.7)	4549 (48.0)
Age (years), Mean ± SD	42.32 ± 15.60	42.35 ± 12.85	42.33 ± 15.33	40.09 ± 15.96	43.39 ± 13.26	41.29 ± 15.12
Han nationality, *n* (%)	8496 (86.5)	1036 (90.1)	9532 (86.9)	5135 (85.0)	3040 (88.3)	8175 (86.2)
Marital status, *n* (%)						
Unmarried	1454 (14.8)	97 (8.5)	1551 (14.1)	1216 (20.2)	260 (7.6)	1476 (15.6)
Married	7693 (78.3)	991 (86.2)	8684 (79.2)	4426 (73.3)	2978 (86.5)	7404 (78.1)
Divorced/separated/widowed	599 (6.1)	51 (4.4)	650 (5.9)	352 (5.8)	171 (5.0)	523 (5.5)
Education level, *n* (%)						
Illiterate	2150 (21.9)	251 (21.9)	2401 (21.9)	1157 (19.2)	877 (25.5)	2034 (21.4)
Primary school	2022 (20.6)	270 (23.5)	2292 (20.9)	1203 (19.9)	806 (23.4)	2009 (21.2)
Middle school	3073 (31.2)	348 (30.3)	3421 (31.2)	2021 (33.5)	1007 (29.2)	3028 (31.9)
High school and above	2377 (24.2)	244 (21.2)	2621 (23.9)	1523 (25.2)	678 (19.7)	2201 (23.2)
Income group, *n* (%)						
Low	4288 (43.6)	511 (44.5)	4799 (43.7)	2710 (44.9)	1582 (45.9)	4292 (45.3)
Medium	3743 (38.1)	463 (40.3)	4206 (38.3)	2272 (37.6)	1368 (39.8)	3640 (38.4)
High	1723 (17.5)	162 (14.1)	1885 (17.2)	1022 (16.9)	459 (13.3)	1481 (15.6)
Community type, *n* (%)						
City	1537 (15.6)	163 (14.2)	1700 (15.5)	1014 (16.8)	491 (14.3)	1505 (15.9)
Suburb	1708 (17.4)	209 (18.2)	1917 (17.5)	1040 (17.2)	613 (17.8)	1653 (17.4)
Town	1785 (18.2)	241 (20.9)	2026 (18.5)	1283 (21.3)	655 (19.0)	1938 (20.4)
Village	4722 (48.1)	564 (49.0)	5286 (48.2)	2679 (44.4)	1673 (48.6)	4352 (45.9)
Regions, n (%)						
Northeast	1890 (19.2)	299 (26.0)	2189 (20.0)	1062 (17.6)	730 (21.2)	1792 (18.9)
East Coast	2097 (21.4)	323 (28.1)	2420 (22.1)	1099 (18.2)	903 (26.2)	2002 (21.1)
Central	3248 (33.1)	360 (31.3)	3608 (32.9)	1975 (32.7)	1163 (33.8)	3138 (33.1)
Western	2586 (26.3)	168 (14.6)	2754 (25.1)	1904 (31.5)	647 (18.8)	2551 (26.9)
Current smoker, n (%)	3082 (31.4)	346 (30.1)	3428 (31.2)	1966 (32.5)	1054 (30.6)	3020 (31.8)
Current drinker, n (%)	3384 (34.5)	399 (34.7)	3783 (34.5)	2074 (34.3)	1211 (35.2)	3285 (34.6)
Physical activities, n (%)						
Light	5778 (58.8)	695 (60.9)	6473 (59.0)	3563 (58.9)	2166 (62.9)	5729 (60.4)
Medium	1355 (13.8)	187 (16.3)	1542 (14.1)	728 (12.1)	556 (16.1)	1284 (13.5)
Heavy	2362 (24.0)	226 (19.7)	2588 (23.6)	1553 (25.7)	609 (17.7)	2162 (22.8)
Dietary total energy intake, kcal/d, Mean ± SD	2259.55 ± 646.14	2325.67 ± 656.75	2266.48 ± 647.55	2259.71 ± 649.11	2301.64 ± 648.52	2274.93 ± 649.17
Meat intake, g/d, Mean ± SD	72.23 ± 88.28	43.88 ± 78.73	69.26 ± 87.76	84.14 ± 89.85	74.22 ± 87.66	80.54 ± 89.19
Dietary total protein intake, g/d, Mean ± SD	68.03 ± 25.77	71.03 ± 27.80	68.34 ± 26.00	67.22 ± 25.03	69.30 ± 26.35	67.98 ± 25.53
History of stroke, n (%)	48 (0.5)	5 (0.4)	53 (0.5)	16 (0.3)	17 (0.5)	33 (0.3)
History of myocardial infarction, n (%)	29 (0.3)	5 (0.4)	34 (0.3)	16 (0.3)	6 (0.2)	22 (0.2)
History of diabetes, n (%)	124 (1.3)	15 (1.3)	139 (1.3)	56 (0.9)	42 (1.2)	98 (1.0)

SD—standard deviation. There were missing data on variables such as marital status, education level, family income, type of community, and physical activity.

**Table 2:** The association between egg consumption and its change trajectory patterns and the risk of obesity: results of Cox proportional hazards regression models with shared frailty.

Egg Consumption	General Obesity				Central Obesity			
	Model 1		Model 2		Model 1		Model 2	
	HR (95% CI)	*p* Value	HR (95% CI)	*p* Value	HR (95% CI)	*p* Value	HR (95% CI)	*p* Value
Egg intake (g/d)								
0.0	1.63 (1.36, 1.94)	<0.001	1.31 (1.08, 1.58)	0.013	1.37 (1.23, 1.54)	<0.001	1.17 (1.04, 1.31)	0.002
0.1~50.0	Reference		Reference		Reference		Reference	
50.1~100.0	1.35 (1.11, 1.65)	0.003	1.30 (1.07, 1.60)	0.021	1.31 (1.14, 1.50)	<0.001	1.31 (1.14, 1.50)	<0.001
>100.0	2.31 (1.38, 3.88)	0.002	1.98 (1.17, 3.35)	0.011	1.78 (1.24, 2.56)	0.002	1.64 (1.15, 2.36)	0.006
Change trajectory patterns of egg intake								
Low Baseline-Stable Pattern	Reference		Reference		Reference		Reference	
Low Baseline-Significant Rising Pattern	1.52 (1.23, 1.87)	<0.001	1.56 (1.25, 1.93)	<0.001	1.44 (1.26, 1.64)	<0.001	1.47 (1.29, 1.68)	<0.001
High Baseline-Rising then Falling Pattern	1.35 (1.15, 1.59)	<0.001	1.38 (1.13, 1.69)	<0.001	1.39 (1.25, 1.54)	<0.001	1.52 (1.34, 1.72)	<0.001

HR—hazard ratio; 95% CI—95% confidence interval. Model 1: the model had the average egg intake, and its change trajectory patterns during the follow-up period, respectively, with the risk factor and family as the random effect. Model 2: further adjusted for sociodemographic factors (including gender, age, nationality, marital status, education levels, family economic level, community type, and region), lifestyle factors (including smoking, drinking, and physical activity), dietary intake (dietary total energy intake and meat intake), history of diseases (including stroke, myocardial infarction, and diabetes) and the baseline year.

**Table 3:** The association between egg-derived cholesterol consumption and its change trajectory patterns and the risk of obesity: results of Cox proportional hazards regression models with shared frailty.

Egg-Derived Cholesterol Consumption	General Obesity				Central Obesity			
	Model 1		Model 2		Model 1		Model 2	
	HR (95% CI)	*p* Value	HR (95% CI)	*p* Value	HR (95% CI)	*p* Value	HR (95% CI)	*p* Value
Quartiles of egg-derived cholesterol intake								
*Q*_1_	1.45 (1.22, 1.74)	<0.001	1.28 (1.06, 1.54)	0.009	1.30 (1.17, 1.45)	<0.001	1.20 (1.08, 1.33)	<0.001
*Q*_2_	Reference		Reference		Reference		Reference	
*Q*_3_	1.24 (1.04, 1.47)	0.014	1.21 (1.02, 1.44)	0.030	1.10 (1.00, 1.22)	0.057	1.11 (1.01, 1.23)	0.038
*Q*_4_	1.49 (1.26, 1.78)	<0.001	1.43 (1.19, 1.71)	<0.001	1.31 (1.19, 1.45)	<0.001	1.32 (1.19, 1.46)	<0.001
Change trajectory patterns of egg-derived cholesterol intake								
Low Baseline-Stable Pattern	Reference		Reference		Reference		Reference	
Low Baseline-Significant Rising Pattern	1.52 (1.23, 1.87)	<0.001	1.54 (1.25, 1.92)	<0.001	1.44 (1.26, 1.64)	<0.001	1.46 (1.28, 1.68)	<0.001
High Baseline-Rising then Falling Pattern	1.35 (1.15, 1.59)	<0.001	1.37 (1.15, 1.64)	<0.001	1.39 (1.25, 1.54)	<0.001	1.47 (1.32, 1.64)	<0.001

HR—hazard ratio; 95% CI—95% confidence interval. The range of egg-derived cholesterol values in each quartile was as follows: Quartile 1: 0 mg/d; Quartile 2: 1.9 to 95.0 mg/d; Quartile 3: 95.5 to 190.1 mg/d; and Quartile 4: 191.0 to 1045.4 mg/d. Model 1: the model had the average egg-derived cholesterol intake and its change trajectory patterns during the follow-up period, respectively, as the risk factor and family as the random effect. Model 2: further adjusted for sociodemographic factors (including gender, age, nationality, marital status, education levels, family economic level, community type, and region), lifestyle factors (including smoking, drinking, and physical activity), dietary intake (dietary total energy intake and dietary total protein intake), history of diseases (including stroke, myocardial infarction, and diabetes), and the baseline year.

## Data Availability

The data were obtained from the CHNS. The original database is available at the website (https://www.cpc.unc.edu/projects/china) (accessed on 16 April 2021).

## Supplementary Materials

The following supporting information can be downloaded at: https://www.mdpi.com/article/10.3390/nu17020333/s1; Table S1: General characteristics of participants in different egg and egg-derived cholesterol intake trajectory groups in the study of general obesity (cohort 1); Table S2: General characteristics of participants in different egg and egg-derived cholesterol intake trajectory groups in the study of central obesity (cohort 2); Table S3: Results of subgroup analyses of the associations of egg consumption with the risk of obesity: Cox proportional hazards regression models with shared frailty; Table S4: Results of subgroup analyses of the associations of egg consumption change trajectory patterns with the risk of obesity: Cox proportional hazards regression models with shared frailty; Table S5: Results of subgroup analyses of the associations of egg-derived cholesterol consumption with the risk of obesity: Cox proportional hazards regression models with shared frailty; Table S6: Results of subgroup analyses of the associations of egg-derived cholesterol consumption change trajectory patterns with the risk of obesity: Cox proportional hazards regression models with shared frailty; Table S7: Results of the sensitivity analyses of the associations of egg consumption and its change trajectory patterns with the risk of obesity: Cox proportional hazards regression models with shared frailty; Table S8: Results of the sensitivity analyses of the associations of egg-derived cholesterol consumption and its change trajectory patterns with the risk of obesity: Cox proportional hazards regression models with shared frailty.
