# Supplementary material for: The Association Between Egg and Egg-Derived Cholesterol Consumption, and Their Change Trajectories, with Obesity Among Chinese Adults: Results from the China Health and Nutrition Survey
Source: Nutrients. 2025 Jan 17;17(2):333. doi: 10.3390/nu17020333 (PMC11767974; doi:10.3390/nu17020333)
Supplement: Supplementary file 1 [file nutrients-17-00333-s001.zip › nutrients-3387841-supplementary.pdf]

The Association Between Egg and Egg-Derived Cholesterol Consumption, and Their Change Trajectories, with Obesity Among Chinese Adults: Results from the China Health and Nutrition Survey

Supplementary Materials

Table S1. General characteristics of participants in different egg and egg-derived cholesterol intake trajectory groups in the study of general obesity (cohort 1).

Characteristics	Low Baseline-Stable Pattern (*n*=3438, 31.3%)	Low Baseline-Significant Rising Pattern (*n*=1715, 15.6%)	High Baseline-Rising then Falling Pattern (*n*=5818, 53.0%)	Total (*n*=10,971)
Duration of follow-up, years, Mean±SD	8.15±5.20	9.30±5.52	7.51±4.43	7.95±4.87
Egg intake, g/d, Mean±SD	7.73±15.01	21.69±20.85	42.57±25.30	22.16±30.15
Egg-derived cholesterol intake, mg/d, Mean±SD	43.18±79.26	122.09±117.18	241.54±143.32	126.39±171.95
Men, *n* (%)	1659 (48.3)	819 (47.8)	2755 (47.4)	5233 (47.7)
Age (years), Mean±SD	42.49±15.79	42.58±13.68	42.15±15.52	42.33±15.33
Han nationality, *n* (%)	2664 (77.5)	1500 (87.5)	5368 (92.3)	9532 (86.9)
Marital status, *n* (%)				
Unmarried	583 (16.9)	157 (9.2)	811 (13.9)	1551 (14.1)
Married	2583 (75.2)	1452 (84.8)	4649 (79.9)	8684 (79.2)
Divorced/separated/widowed	246 (7.1)	92 (5.4)	312 (5.4)	650 (5.9)
Education level, *n* (%)				
Illiterate	1025 (29.8)	430 (25.1)	946 (16.3)	2401 (21.9)
Primary school	806 (23.4)	434 (25.4)	1052 (18.1)	2292 (20.9)
Middle school	1026 (29.9)	532 (31.0)	1863 (32.0)	3421 (31.2)
High school and above	507 (14.7)	281 (16.4)	1833 (31.5)	2621 (23.9)
Income group, *n* (%)				
Low	1154 (33.6)	478 (27.9)	3167 (54.4)	4799 (43.7)
Medium	1301 (37.9)	656 (38.3)	2249 (38.7)	4206 (38.3)
High	968 (28.2)	291 (16.9)	626 (10.8)	1885 (17.2)
Community type, *n* (%)				
City	409 (11.9)	190 (11.1)	1101 (18.9)	1700 (15.5)
Suburb	401 (11.6)	224 (13.1)	1292 (22.2)	1917 (17.5)

Continued Table S1

Characteristics	Low Baseline-Stable Pattern (*n*=3438, 31.3%)	Low Baseline-Significant Rising Pattern (*n*=1715, 15.6%)	High Baseline-Rising then Falling Pattern (*n*=5818, 53.0%)	Total (*n*=10,971)
Town	724 (21.1)	309 (18.0)	993 (17.1)	2026 (18.5)
Village	1797 (52.3)	954 (55.6)	2535 (43.5)	5286 (48.2)
Regions, *n* (%)				
Northeast	388 (11.3)	376 (21.9)	1425 (24.5)	2189 (20.0)
East Coast	434 (12.6)	346 (20.2)	1640 (28.2)	2420 (22.1)
Central	996 (29.0)	546 (31.8)	2066 (35.5)	3608 (32.9)
Western	1620 (47.1)	447 (26.1)	687 (11.8)	2754 (25.1)
Current smoker, *n* (%)	1151 (33.5)	556 (32.4)	1721 (29.6)	3428 (31.2)
Current drinker, *n* (%)	1190 (34.6)	563 (32.8)	2030 (34.9)	3783 (34.5)
Physical activities, *n* (%)				
Light	2397 (69.7)	923 (53.9)	3153 (54.2)	6473 (59.0)
Medium	322 (9.4)	179 (10.4)	1041 (17.9)	1542 (14.1)
Heavy	610 (17.7)	477 (27.8)	1501 (25.8)	2588 (23.6)
Dietary total energy intake, kcal/d. Mean±SD	2322.16±676.51	2296.15±653.36	2224.82±625.10	2266.48±647.55
Meat intake, g/d. Mean±SD	67.12±87.66	64.47±87.68	74.20±87.67	69.26±87.76
Dietary total protein intake, g/d. Mean±SD	66.36±25.35	67.09±27.16	69.88±25.94	68.34±26.00
History of diseases, *n* (%)				
Stroke	10 (0.3)	7 (0.4)	36 (0.6)	53 (0.5)
Myocardial infarction	11 (0.3)	3 (0.2)	20 (0.3)	34 (0.3)
Diabetes	31 (0.9)	17 (1.0)	91 (1.6)	139 (1.3)
General obesity, *n* (%)	291 (8.5)	220 (12.8)	639 (11.0)	1150 (10.5)

SD—standard deviation. There were missing data on variables such as marital status, education level, family income, type of community and physical activity.

Table S2. General characteristics of participants in different egg and egg-derived cholesterol intake trajectory groups in the study of central obesity (cohort 2).

Characteristics	Low Baseline-Stable Pattern (*n*=3102, 32.7%)	Low Baseline-Significant Rising Pattern (*n*=1530, 16.1%)	High Baseline-Rising then Falling Pattern (*n*=4851, 51.2%)	Total (*n*=9483)
Duration of follow-up, years, Mean±SD	7.12±4.69	7.78±4.99	6.38±3.91	6.82±4.37
Egg intake, g/d, Mean±SD	7.39±14.97	19.50±21.23	41.72±25.62	20.61±29.02
Egg-derived cholesterol intake, mg/d, Mean±SD	41.15±78.34	109.53±119.12	236.72±145.11	117.52±165.46
Men, *n* (%)	1527 (49.2)	737 (48.2)	2285 (47.1)	4549 (48.0)
Age (years), Mean±SD	41.84±15.73	41.95±13.60	40.73±15.16	41.29±15.12
Han nationality, *n* (%)	2384 (76.9)	1340 (87.6)	4451 (91.8)	8175 (86.2)
Marital status, *n* (%)				
Unmarried	557 (17.9)	153 (10.0)	766 (15.8)	1476 (15.6)
Married	2300 (74.1)	1290 (84.3)	3814 (78.5)	7404 (78.0)
Divorced/separated/widowed	215 (6.9)	75 (4.9)	233 (4.8)	523 (5.6)
Education level, *n* (%)				
Illiterate	896 (28.9)	377 (24.6)	761 (15.7)	2034 (21.4)
Primary school	740 (23.9)	393 (25.7)	876 (18.1)	2009 (21.2)
Middle school	947 (30.5)	476 (31.1)	1605 (33.1)	3028 (31.9)
High school and above	451 (14.5)	249 (16.3)	1501 (30.9)	2201 (23.2)
Income group, *n* (%)				
Low	1403 (45.2)	691 (45.1)	2198 (45.3)	4292 (45.3)
Medium	1190 (38.3)	467 (30.5)	1983 (40.9)	3640 (38.4)
High	501 (16.1)	271 (17.7)	709 (14.6)	1481 (15.7)
Community type, *n* (%)				
City	353 (11.4)	178 (11.6)	974 (20.1)	1505 (15.9)
Suburb	430 (13.9)	202 (13.2)	1021 (21.0)	1653 (17.4)

Continued Table S2

Characteristics	Low Baseline-Stable Pattern (*n*=3102, 32.7%)	Low Baseline-Significant Rising Pattern (*n*=1530, 16.1%)	High Baseline-Rising then Falling Pattern (*n*=4851, 51.2%)	Total (*n*=9483)
Town	668 (21.5)	378 (24.7)	892 (18.4)	1938 (20.4)
Village	1537 (49.5)	749 (48.9)	2067 (42.6)	4352 (45.9)
Regions, *n* (%)				
Northeast	333 (10.7)	313 (20.5)	1146 (23.6)	1792 (18.9)
East Coast	379 (12.2)	304 (19.9)	1319 (27.2)	2002 (21.1)
Central	876 (28.2)	495 (32.4)	1767 (36.4)	3138 (33.1)
Western	1514 (48.8)	418 (27.3)	619 (12.8)	2551 (26.9)
Current smoker, *n* (%)	1061 (34.2)	506 (33.1)	1453 (30.0)	3020 (31.8)
Current drinker, *n* (%)	1097 (35.4)	495 (32.4)	1693 (34.9)	3285 (34.6)
Physical activities, *n* (%)				
Light	1873 (60.4)	922 (60.2)	2934 (60.5)	5729 (60.4)
Medium	401 (12.9)	217 (14.2)	666 (13.7)	1284 (13.5)
Heavy	819 (26.4)	348 (22.7)	995 (20.5)	2162 (22.8)
Dietary total energy intake, kcal/d, Mean±SD	2333.13±675.92	2297.40±657.41	2230.63±625.50	2274.93±649.17
Meat intake, g/d, Mean±SD	75.50±89.56	74.70±89.81	89.18±87.75	80.54±89.19
Dietary total protein intake, g/d, Mean±SD	66.31±24.74	66.68±26.97	69.45±25.48	67.98±25.53
History of diseases, *n* (%)				
Stroke	28 (0.9)	12 (0.8)	58 (1.2)	98 (1.0)
Myocardial infarction	9 (0.3)	2 (0.1)	11 (0.2)	22 (0.2)
Diabetes	8 (0.3)	5 (0.3)	20 (0.4)	33 (0.3)
Central obesity, *n* (%)	950 (30.6)	667 (43.6)	1826 (37.6)	3443 (31.0)

SD—standard deviation. There were missing data on variables such as marital status, education level, family income, type of community and physical activity.

Table S3. Results of subgroup analyses of the associations of egg consumption with the risk of obesity: Cox proportional hazards regression models with shared frailty.

Subgroups	HR (95% CI) for general obesity				*p* for interaction	HR (95% CI) for central obesity				*p* for interaction
	Egg consumption					Egg consumption				
	0.0 g/d	0.1~50.0 g/d	50.1~100.0 g/d	>100.0 g/d		0.0 g/d	0.1~50.0 g/d	50.1~100.0 g/d	>100.0 g/d	
Gender										
Men	1.02 (1.01, 1.24)	Reference	1.28 (1.04, 1.75)	2.05 (1.37, 3.08)	0.064	1.01 (1.00, 1.34)	Reference	1.08 (0.88, 1.34)	1.32 (0.96, 1.81)	0.067
Women	1.16 (1.12, 1.24)	Reference	1.04 (1.01, 1.43)	1.95 (1.21, 3.16)		1.06 (1.03, 1.47)	Reference	1.23 (0.76, 1.34)	1.88 (1.41, 2.51)	
Age										
<65	0.89 (0.73, 1.08)	Reference	1.11 (0.84, 1.47)	2.13 (1.45, 3.13)	0.434	1.00 (0.89, 1.12)	Reference	1.01 (0.84, 1.21)	1.03 (0.79, 1.33)	0.167
≥65	1.14 (0.43, 1.96)	Reference	1.21 (0.70, 2.09)	2.24 (1.03, 4.90)		1.00 (0.80, 1.24)	Reference	0.97 (0.70, 1.34)	1.19 (0.79, 1.79)	
Smoking										
Current	1.16 (0.90, 1.49)	Reference	1.50 (1.10, 2.61)	1.98 (1.40, 2.80)	0.053	1.12 (0.56, 2.24)	Reference	1.18 (0.41, 3.43)	1.92 (1.21, 2.67)	0.091
Never	0.84 (0.71, 1.01)	Reference	1.12 (1.03, 1.45)	1.54 (1.34, 1.89)		0.99 (0.89, 1.09)	Reference	1.00 (0.85, 1.17)	0.98 (0.79, 1.22)	
Drinking										
Current	1.28 (0.80, 2.05)	Reference	1.28 (1.00, 1.44)	1.33 (0.71, 1.37)	0.053	1.07 (0.80, 1.28)	Reference	1.26 (1.08, 1.70)	1.41 (0.71, 1.81)	0.431
Never	1.11 (0.99, 1.24)	Reference	1.07 (1.02, 1.21)	1.13 (1.00, 1.27)		1.10 (0.98, 1.23)	Reference	1.07 (0.95, 1.20)	1.12 (1.00, 1.27)	
Physical activities										
Light	1.02 (0.78, 1.36)	Reference	1.10 (0.74, 1.63)	1.79 (1.10, 6.12)	0.433	1.03 (0.79, 1.08)	Reference	0.97 (0.76, 1.24)	1.13 (0.81, 1.57)	0.556
Medium	0.96 (0.71, 1.31)	Reference	1.35 (0.91, 1.99)	2.00 (0.79, 5.02)		1.08 (0.90, 1.29)	Reference	1.03 (0.78, 1.37)	1.29 (0.86, 1.32)	
Heavy	1.11 (0.51, 1.38)	Reference	1.22 (0.79, 1.88)	1.10 (0.25, 4.87)		0.96 (0.78, 1.19)	Reference	1.06 (0.78, 1.44)	1.60 (0.99, 2.59)	

HR—Hazard ratio; 95% CI—95% confidence interval. This model had the average egg intake during the follow-up period as the risk factor and family as the random effect, and adjusted for sociodemographic factors (including gender, age, nationality, marital status, education levels, family economic level, community type and region), lifestyle factors (including smoking, drinking and physical activity), dietary intake (dietary total energy intake and meat intake), history of diseases (including stroke, myocardial infarction and diabetes) and baseline year, excluding the stratified variable.

Table S4. Results of subgroup analyses of the associations of egg consumption change trajectory patterns with the risk of obesity: Cox proportional hazards regression models with shared frailty.

Subgroups	HR (95% CI) for general obesity			*p* for interaction	HR (95% CI) for central obesity			*p* for interaction
	Egg consumption change trajectory patterns				Egg consumption change trajectory patterns			
	Low Baseline-Stable Pattern	Low Baseline-Significant Rising Pattern	High Baseline-Rising then Falling Pattern		Low Baseline-Stable Pattern	Low Baseline-Significant Rising Pattern	High Baseline-Rising then Falling Pattern	
Gender								
Men	Reference	1.83 (1.34, 2.49)	1.99 (1.59, 2.49)	0.267	Reference	1.83 (1.34, 2.49)	1.80 (1.32, 2.46)	0.516
Women	Reference	1.80 (1.32, 2.46)	1.98 (1.57, 2.51)		Reference	1.99 (1.59, 2.49)	1.98 (1.57, 2.51)	
Age								
<65	Reference	1.43 (1.14, 1.79)	1.15 (0.96, 1.37)	0.126	Reference	1.41 (1.12, 1.77)	1.15 (0.95, 1.38)	0.748
≥65	Reference	1.53 (1.23, 1.87)	1.27 (1.15, 1,89)		Reference	1.48 (1.17, 1.86)	1.27 (1.02, 1.59)	
Smoking								
Current	Reference	1.58 (1.32, 1.90)	1.70 (1.49, 1.94)	0.321	Reference	1.31 (1.15, 1.49)	1.32 (1.16, 1.50)	0.356
Never	Reference	1.47 (1.23, 1.77)	1.55 (1.35, 1.78)		Reference	1.19 (1.07, 1.31)	1.32 (1.19, 1.47)	
Drinking								
Current	Reference	1.58 (1.32, 1.90)	1.70 (1.49, 1.94)	0.073	Reference	1.41 (1.12, 1.78)	1.43 (1.14, 1.79)	0.423
Never	Reference	1.51 (1.26, 1.82)	1.65 (1.41, 1.93)		Reference	1.12 (0.93, 1.35)	1.15 (0.96, 1.37)	
Physical activities								
Light	Reference	1.36 (1.19, 1.55)	1.32 (1.16, 1.51)	0.281	Reference	1.48 (1.23, 1.78)	1.43 (1.14, 1.79)	0.067
Medium	Reference	1.44 (1.27, 1.63)	1.31 (1.18, 1.46)		Reference	1.57 (1.37, 1.81)	1.15 (0.96, 1.37)	
Heavy	Reference	1.58 (1.32, 1.90)	1.51 (1.26, 1.82)		Reference	1.45 (1.32, 1.90)	1.48 (1.23, 1.78)	

HR—Hazard ratio; 95% CI—95% confidence interval. This model had the egg intake change trajectory patterns during the follow-up period as the risk factor and family as the random effect, and adjusted for sociodemographic factors (including gender, age, nationality, marital status, education levels, family economic level, community type and region), lifestyle factors (including smoking, drinking and physical activity), dietary intake (dietary total energy intake and meat intake), history of diseases (including stroke, myocardial infarction and diabetes) and baseline year, excluding the stratified variable.

Table S5. Results of subgroup analyses of the associations of egg-derived cholesterol consumption with the risk of obesity: Cox proportional hazards regression models with shared frailty.

Subgroups	HR (95% CI) for general obesity				p for interaction	HR (95% CI) for central obesity				p for interaction
	Egg-derived cholesterol consumption					Egg-derived cholesterol consumption				
	Q_1	Q_2	Q_3	Q_4		Q_1	Q_2	Q_3	Q_4	
Gender										
Men	1.07 (0.66, 1.15)	Reference	1.09 (1.05, 1.31)	1.29 (1.08, 1.70)	0.052	1.09 (0.92, 1.28)	Reference	1.12 (1.03, 1.17)	1.16 (1.04, 1.37)	0.163
Women	0.90 (0.56, 1.16)	Reference	1.03 (0.58, 1.91)	1.21 (0.99, 1.91)		1.03 (0.73, 1.54)	Reference	1.16 (0.75, 1.47)	1.09 (0.77, 1.42)	
Age										
<65	1.05 (0.61, 1.33)	Reference	1.10 (1.02, 1.58)	1.20 (0.73, 1.41)	0.754	1.17 (1.03, 1.33)	Reference	1.11 (0.98, 1.27)	1.18 (1.03, 1.36)	0.262
≥65	1.27 (1.07, 1.89)	Reference	1.35 (1.42, 1.80)	1.48 (1.23, 1.78)		1.25 (1.04, 1.31)	Reference	1.29 (1.08, 1.66)	1.34 (1.02, 1.45)	
Smoking										
Current	1.37(1.07, 1.54)	Reference	1.12(0.96, 1.31)	2.12(1.43, 3.21)	0.256	0.96 (0.80, 1.15)	Reference	0.98 (0.83, 1.16)	1.26 (1.06, 1.49)	0.072
Never	1.17(1.03, 1.36)	Reference	1.10(0.95, 1.28)	1.92(1.25, 2.82)		1.18 (1.03, 1.34)	Reference	1.12 (0.99, 1.27)	1.27 (1.11, 1.45)	
Drinking										
Current	1.12 (1.02, 1.39)	Reference	1.21 (1.09, 1.67)	1.25 (1.07, 1.37)	0.472	1.06 (0.93, 1.20)	Reference	1.12 (0.99, 1.27)	1.15 (1.01, 1.31)	0.863
Never	1.03 (1.00, 1.45)	Reference	1.18 (1.12, 1.76)	1.23 (1.14, 1.56)		1.17 (1.03, 1.33)	Reference	1.27 (1.11, 1.45)	1.32 (1.11, 1.56)	
Physical activities										
Light	1.13 (1.04, 1.89)	Reference	1.18 (1.08, 1.97)	1.22 (1.02, 1.45)	0.451	1.23 (1.06, 1.44)	Reference	1.22 (0.96, 1.42)	1.39 (1.00, 1.72)	0.054
Medium	1.09 (0.97, 1.18)	Reference	1.04 (1.01, 1.25)	1.10 (1.03, 1.21)		1.16 (1.07, 1.74)	Reference	1.27 (1.12, 1.78)	1.38 (1.02, 1.49)	
Heavy	1.17 (1.07, 1.97)	Reference	1.23 (1.11, 1.95)	1.27 (1.07, 1.41)		1.30 (0.98, 1.48)	Reference	1.41 (1.03, 1.63)	1.49 (1.29, 1.85)	

HR—Hazard ratio; 95% CI—95% confidence interval. Range of egg-derived cholesterol values in each quartile was as follows: Quartile 1: 0 mg/d; Quartile 2: 1.9 to 95.0 mg/d; Quartile 3: 95.5 to 190.1 mg/d; Quartile 4: 191.0 to 1045.4 mg/d. The model had the average egg-derived cholesterol intake during the follow-up period as the risk factor and family as the random effect, adjusted for sociodemographic factors (including gender, age, nationality, marital status, education levels, family economic level, community type and region), lifestyle factors (including smoking, drinking and physical activity), dietary intake (dietary total energy intake and dietary total protein intake), history of diseases (including stroke, myocardial infarction and diabetes) and baseline year, excluding the stratified variable.

Table S6. Results of subgroup analyses of the associations of egg-derived cholesterol consumption change trajectory patterns with the risk of obesity: Cox proportional hazards regression models with shared frailty.

Subgroups	HR (95% CI) for general obesity			*p* for interaction	HR (95% CI) for central obesity			*p* for interaction
	Egg-derived cholesterol consumption pattern				Egg-derived cholesterol consumption pattern			
	Low Baseline-Stable Pattern	Low Baseline-Significant Rising Pattern	High Baseline-Rising then Falling Pattern		Low Baseline-Stable Pattern	Low Baseline-Significant Rising Pattern	High Baseline-Rising then Falling Pattern	
Gender								
Men	Reference	1.30 (1.01, 1.67)	1.47 (1.26, 1.73)	0.136	Reference	1.29 (1.06, 1.86)	1.10 (0.97, 1.24)	0.236
Women	Reference	1.32 (1.07, 1.62)	1.32 (1.16, 1.50)		Reference	1.39 (1.26, 1.47)	1.37 (1.21, 1.76)	
Age								
<65	Reference	1.15 (0.95, 1.38)	1.27 (1.02, 1.59)	0.059	Reference	1.55 (1.25, 1.93)	1.38 (1.16, 1.65)	0.705
≥65	Reference	1.41 (1.12, 1.77)	1.48 (1.17, 1.86)		Reference	1.54 (1.25, 1.92)	1.37 (1.15, 1.64)	
Smoking								
Current	Reference	1.15 (0.96, 1.37)	1.48 (1.37, 2.51)	0.214	Reference	1.58 (1.32, 1.90)	1.70 (1.49, 1.94)	0.174
Never	Reference	1.43 (1.14, 1.79)	1.80 (1.32, 2.46)		Reference	1.52 (1.23, 1.87)	1.35 (1.15, 1.59)	
Drinking								
Current	Reference	1.43 (1.14, 1.79)	1.41 (1.12, 1.78)	0.078	Reference	1.56 (1.25, 1.93)	1.38 (1.13, 1.69)	0.061
Never	Reference	1.15 (0.96, 1.37)	1.12 (0.93, 1.35)		Reference	1.44 (1.26, 1.64)	1.39 (1.25, 1.54)	
Physical activities								
Light	Reference	1.41 (1.12, 1.77)	1.51 (1.26, 1.82)	0.405	Reference	1.47 (1.29, 1.68)	1.46 (1.28, 1.67)	0.475
Medium	Reference	1.15 (0.95, 1.38)	1.17 (1.01, 1.93)		Reference	1.52 (1.34, 1.72)	1.48 (1.33, 1.65)	
Heavy	Reference	1.31 (1.15, 1.49)	1.19 (1.07, 1.31)		Reference	1.46 (1.28, 1.68)	1.47 (1.32, 1.64)	

HR—Hazard ratio; 95% CI—95% confidence interval. The model had the egg-derived cholesterol intake change trajectory patterns during the follow-up period as the risk factor and family as the random effect, and adjusted for sociodemographic factors (including gender, age, nationality, marital status, education levels, family economic level, community type and region), lifestyle factors (including smoking, drinking and physical activity), dietary intake (dietary total energy intake and dietary total protein intake), history of diseases (including stroke, myocardial infarction and diabetes) and baseline year, excluding the stratified variable.

Table S7. Results of the sensitivity analyses of the associations of egg consumption and its change trajectory patterns with the risk of obesity: Cox proportional hazards regression models with shared frailty.

Egg consumption	General obesity				Central obesity			
	Model 1		Model 2		Model 1		Model 2	
	HR (95% CI)	*p* value	HR (95% CI)	*p* value	HR (95% CI)	*p* value	HR (95% CI)	*p* value
Among participants with at least 3 dietary measurements								
Egg intake (g/d)								
0.0	1.84 (1.54, 2.20)	<0.001	1.32 (1.08, 1.60)	0.008	1.62 (1.45, 1.81)	<0.001	1.24 (1.10, 1.39)	<0.001
0.1~50.0	Reference		Reference		Reference		Reference	
50.1~100.0	1.53 (1.21, 1.94)	<0.001	1.37 (1.10, 1.70)	0.003	1.38 (1.19, 1.59)	<0.001	1.40 (1.22, 1.61)	<0.001
>100.0	1.95 (0.78, 4.85)	0.150	2.17 (1.23, 3.84)	0.005	2.19 (1.46, 3.29)	<0.001	1.88 (1.26, 2.81)	0.002
Trajectory patterns of egg intake								
Low Baseline-Stable Pattern	Reference		Reference		Reference		Reference	
Low Baseline-Significant Rising Pattern	1.30 (1.01, 1.67)	0.044	1.32 (1.01, 1.71)	0.039	1.35 (1.16, 1.57)	<0.001	1.35 (1.16, 1.58)	<0.001
High Baseline-Rising then Falling Pattern	1.32 (1.07, 1.62)	0.009	1.35 (1.04, 1.74)	0.025	1.39 (1.22, 1.58)	<0.001	1.48 (1.27, 1.73)	<0.001
Among participants with missing values in covariates imputed								
Egg intake (g/d)								
0.0	1.60 (1.23, 1.82)	<0.001	1.35 (1.10, 1.65)	0.023	1.35 (1.20, 1.62)	0.002	1.20 (1.02, 1.42)	0.002
0.1~50.0	Reference		Reference		Reference		Reference	
50.1~100.0	1.30 (1.21, 1.77)	0.005	1.34 (1.07, 1.70)	0.016	1.28 (1.11, 1.50)	<0.001	1.31 (1.10, 1.50)	<0.001
>100.0	2.01 (1.28, 2.59)	0.004	1.89 (1.20, 3.42)	0.013	1.80 (1.32, 2.70)	0.018	1.64 (1.15, 2.46)	0.003
Trajectory patterns of egg intake								
Low Baseline-Stable Pattern	Reference		Reference		Reference		Reference	
Low Baseline-Significant Rising Pattern	1.32 (1.13, 1.87)	<0.001	1.60 (1.25, 1.99)	<0.001	1.44 (1.30, 1.64)	<0.001	1.45 (1.30, 1.68)	<0.001
High Baseline-Rising then Falling Pattern	1.42 (1.20, 1.62)	<0.001	1.34 (1.14, 1.69)	<0.001	1.40 (1.25, 1.64)	<0.001	1.52 (1.26, 1.80)	<0.001

HR—Hazard ratio; 95% CI—95% confidence interval. Model 1: This model had the average egg intake and its change trajectory patterns during the follow-up period, respectively, as the risk factor and family as the random effect. Model 2: Further adjusted for sociodemographic factors (including gender, age, nationality, marital status, education levels, family economic level, community type and region), lifestyle factors (including smoking, drinking and physical activity), dietary intake (dietary total energy intake and meat intake), history of diseases (including stroke, myocardial infarction and diabetes) and baseline year.

Table S8. Results of the sensitivity analyses of the associations of egg-derived cholesterol consumption and its change trajectory patterns with the risk of obesity: Cox proportional hazards regression models with shared frailty.

Egg-derived cholesterol consumption	General obesity				Central obesity			
	Model 1		Model 2		Model 1		Model 2	
	HR (95% CI)	*p* value	HR (95% CI)	*p* value	HR (95% CI)	*p* value	HR (95% CI)	*p* value
Among participants with at least 3 dietary measurements								
Quartiles of egg-derived cholesterol intake								
Q_1	1.58 (1.30, 1.93)	<0.001	1.33 (1.09, 1.64)	0.006	1.48 (1.32, 1.66)	<0.001	1.29 (1.15, 1.46)	<0.001
Q_2	Reference		Reference		Reference		Reference	
Q_3	1.24 (1.02, 1.50)	0.030	1.23 (1.01, 1.49)	0.037	0.98 (0.88, 1.10)	0.785	0.98 (0.88, 1.10)	0.729
Q_4	1.46 (1.20, 1.77)	<0.001	1.45 (1.19, 1.77)	<0.001	1.23 (1.10, 1.38)	<0.001	1.26 (1.13, 1.42)	<0.001
Trajectory patterns of egg-derived cholesterol intake								
Low Baseline-Stable Pattern	Reference		Reference		Reference		Reference	
Low Baseline-Significant Rising Pattern	1.30 (1.01, 1.67)	0.044	1.32 (1.02, 1.71)	0.037	1.35 (1.16, 1.57)	<0.001	1.35 (1.15, 1.57)	<0.001
High Baseline-Rising then Falling Pattern	1.32 (1.07, 1.62)	0.009	1.33 (1.06, 1.66)	0.012	1.39 (1.22, 1.58)	<0.001	1.43 (1.25, 1.64)	<0.001
Among participants with missing values in covariates imputed								
Quartiles of egg-derived cholesterol intake								
Q_1	1.42 (1.15, 1.84)	0.002	1.27 (1.10, 1.64)	0.005	1.28 (1.05, 1.65)	<0.001	1.18 (1.02, 1.52)	<0.001
Q_2	Reference		Reference		Reference		Reference	
Q_3	1.30 (1.08, 1.53)	0.037	1.20 (1.05, 1.52)	0.038	1.20 (1.05, 1.72)	0.056	1.11 (1.02, 1.73)	0.042
Q_4	1.45 (1.20, 1.80)	<0.001	1.33 (1.02, 1.78)	<0.001	1.28 (1.20, 1.65)	<0.001	1.32 (1.15, 1.58)	<0.001
Trajectory patterns of egg-derived cholesterol intake								
Low Baseline-Stable Pattern	Reference		Reference		Reference		Reference	
Low Baseline-Significant Rising Pattern	1.48 (1.20, 1.85)	<0.001	1.46 (1.12, 1.82)	<0.001	1.40 (1.26, 1.74)	<0.001	1.46 (1.08, 1.62)	<0.001
High Baseline-Rising then Falling Pattern	1.35 (1.05, 1.64)	<0.001	1.38 (1.05, 1.74)	<0.001	1.35 (1.05, 1.78)	<0.001	1.38 (1.02, 1.64)	<0.001

HR—Hazard ratio; 95% CI—95% confidence interval. Range of egg-derived cholesterol values in each quartile was as follows: Quartile 1: 0 mg/d; Quartile 2: 1.9 to 95.0 mg/d; Quartile 3: 95.5 to 190.1 mg/d; Quartile 4: 191.0 to 1045.4 mg/d. Model 1: The model had the average egg-derived cholesterol intake and its change trajectory patterns during the follow-up period, respectively, as the risk factor and family as the random effect. Model 2: Further adjusted for sociodemographic factors (including gender, age, nationality, marital status, education levels, family economic level, community type and region), lifestyle factors (including smoking, drinking and physical activity), dietary intake (dietary total energy intake and dietary total protein intake), history of diseases (including stroke, myocardial infarction and diabetes) and baseline year.
